# Circ_0023404 sponges miR‐136 to induce HK‐2 cells injury triggered by hypoxia/reoxygenation via up‐regulating IL‐6R

**DOI:** 10.1111/jcmm.15986

**Published:** 2021-05-04

**Authors:** Yong Xu, Xiang Li, Hailun Li, Lili Zhong, Yongtao Lin, Juan Xie, Donghui Zheng

**Affiliations:** ^1^ Department of Nephrology Affiliated Huai’an Hospital of Xuzhou Medical University Huai’an China

**Keywords:** acute kidney injury, Circ_0023404, Hypoxia/Reoxygenation, IL‐6R, miR‐136

## Abstract

The significance of circular RNAs (circRNAs) is reported in various kidney diseases including acute kidney injury (AKI). Specific circRNAs have the capacity to function as novel indicators of AKI. Circ_0023404 exhibits an important role in several diseases. Nevertheless, the detailed biological role of circ_0023404 in AKI remains poorly known. The present study aimed to investigate the effect of circ_0023404 on renal ischaemia/reperfusion (I/R) injury in vitro. Here, we evaluated the function of circ_0023404 in HK‐2 cells in response to hypoxia/reoxygenation (H/R). We established a cell AKI model induced by H/R in HK‐2 cells. We found circ_0023404 was significantly increased in AKI. Then, we found loss of circ_0023404 increased cell growth, repressed apoptosis, reduced inflammatory factors secretion and oxidative stress generation in vitro. Besides, circ_0023404 sponged miR‐136. miR‐136 overturned the effects of circ_0023404 on HK‐2 cell injury. We assumed IL‐6 receptor (IL‐6R) as a target of miR‐136 and IL‐6R was activated by circ_0023404 via sponging miR‐136. In conclusion, we revealed circ_0023404 contributed to HK‐2 cells injury stimulated by H/R via sponging miR‐136 and activating IL‐6R.

## 1 INTRODUCTION

Acute kidney injury (AKI) is a syndrome with degradation of renal function.^1^ AKI is becoming a serious public health problem across the world.^2^ Risk factors such as inflammation, overdose of drug and traumatism can all contribute to AKI progression.^3^, ^4^ Additionally, during the process of transplantation of kidney, ischaemia/reperfusion (I/R) can lead to AKI.^5^, ^6^ I/R injury is resulted from the massive oxygen and nutrients transmitted to kidney epithelial cells manifesting apoptosis, inflammatory response and necrosis. Consequently, increasing researchers focus on the anti‐inflammatory and anti‐oxidant agents to develop a new medicine for renal I/R injury. However, the available therapy for renal I/R injury still remains very limited.

An increasing number of reports have displayed non‐coding RNAs are closely involved in many diseases, including circRNAs, lncRNAs and microRNAs.^7^ CircRNAs are a kind of novel non‐coding RNA with closed structure.^8^ Because of the high stability and conservatism of circRNAs, the functions of circRNAs have been identified and investigated by more scholars in different diseases.^9^ Recently, circRNAs are reported to be implicated in a wide range of diseases including AKI progression.^10^ For example, circular RNA can exhibit a protective effect on AKI model.^11^ Circ‐ciRs‐126 can indicate survival in critically Ill AKI patients.^12^ CircPRKCI can reduce LPS‐triggered HK2 cell injury through inducing the zinc finger E‐box‐binding homeobox 1 (ZEB2) targeted by miR‐545.^13^ Additionally, circRNAs are shown to function as sinks for miRNAs and they can control the function of miRNAs.^14^ However, it is unknown whether circ_0023404 plays an important influence in AKI and little is reported about its mechanisms.

In the work, we established H/R model using HK‐2 cells. We tested expression level of circ_0023404 in AKI patients and HK‐2 cells treated with H/R condition. Then, we studied the effects of circ_0023404 on cell proliferation, apoptosis, inflammatory response and oxidative stress. Meanwhile, miR‐136 acted as a downstream target of circ_0023404 and IL‐6R acted as a target of miR‐136. miRt136 has been experimentally verified as new biomarkers in various diseases, including renal diseases.^15^ Therefore, it is suggested that miR‐136 may exhibit great promise as a crucial biomarker for AKI. We explored the role of circ_0023404/miR‐136/IL‐6R axis in the development of AKI. This study might indicate that circ_0023404 can function as a novel therapeutic target for AKI via modulating miR‐136 and IL‐6R.

## 2 MATERIALS AND METHODS

### 2.1 Patient samples

The serum samples of 30 AKI cases and aged‐equal healthy cases were attained from Affiliated Huai'an Hospital of Xuzhou Medical University. No patients had been given any therapies before enrolment. We obtained the informed consents and medical ethics certification from the Medical Ethics Committee of Affiliated Huai'an Hospital of Xuzhou Medical University. Venous blood was disposed within 1 hour after collection. In brief, serum samples were isolated at centrifugation of 1200 *g* for 10 minutes. Then, another centrifugation (10 000 *g*, 10 minutes) was followed to discard residual cellular debris. All centrifugations were carried out at 4°C. All serum samples were maintained in liquid nitrogen for RNA extraction.

### 2.2 Cells

HK‐2 cells were purchased from Shanghai Institutes for Biological Sciences (Shanghai, China). We cultured the cells using DMEM added with 10% FBS and 1% penicillin and streptomycin under a humidified atmosphere consisting of 5% CO_2_ and 95% air at 37°C (control group). Hypoxia and reoxygenation (H/R) injury was induced by exposing HK‐2 cells to hypoxic conditions (1% O_2_, 5% CO_2_ and 94% N_2_) for 12 hours, followed by reoxygenation for 2 hours in fresh normal medium (H/R group).

### 2.3 Cell transfection

miR‐136 mimics and their NC controls were obtained from GenePharma (Shanghai, China). Lipofectamine 3000 (Invitrogen, Carlsbad, CA, USA) was utilized to carry out transfection. The overexpressing circ_0023404 plasmids and siRNA of circ_0023404 were colligated by Sangon (Shanghai, China). The target sequence of si‐circ_0023404‐01 (5′‐ACGCTCCTACAATGTTGATAT‐3′), si‐circ_0023404‐02 (5′‐GCTCCTACAATGTTGATATGT‐3′) and si‐circ_0023404‐03 (5′‐CACGCTCCTACAATGTTGATA‐3′) was exhibited.

### 2.4 CCK‐8 assay

After transfection, 2 × 10^3^ cells were seeded in a 96‐well plate, containing 10% FBS. 10 μL CCK‐8 solution (Beyotime, Shanghai, China) was added to the cells for 2 hours in a 5% CO_2_ incubator at 37°C. A spectrophotometer (Bio‐Rad, Hercules, CA, USA) was conducted to record the absorbance at 450 nm.

### 2.5 Cell apoptosis assay

Cell apoptosis was determined by Hoechst 33258 staining assay (Beyotime, Shanghai, China). At 48 hours after transfection, apoptotic cells were incubated with Hoechst 33342 at 4°C for 10 minutes, and then, a fluorescence microscope was used to determine the apoptosis.

### 2.6 qRT‐PCR

The total RNA of the transfected cells and serum samples was extracted using the TRIzol Reagent (Invitrogen, Carlsbad, CA, USA). Then, concentration and purity of the total RNA were analysed using UV spectrophotometer analysis at 260 nm. cDNA Reverse Transcription Kit (Ambion, Austin, TX, USA) was used to do reverse transcription. Next, qPCR analysis was conducted utilizing SYBR^®^ Green PCR Kit (TaKaRa, Dalian, China) on iQ5 Real‐Time PCR amplification (Applied Biosystems, Foster City, CA, USA) according to the instructions. All calculation was carried out according to 2^−ΔΔCt^ methods. Primers for qPCR analysis are displayed in Table 1.

**TABLE 1:** Primers used for real‐time PCR

Genes	Forward (5’‐3’)	Reverse (5’‐3’)
GAPDH	TGTTGCCATCAATGACCCCTT	CTCCACGACGTACTCAGCG
U6	CTCGCTTCGGCAGCACA	AACGCTTCACGAATTTGCGT
Circ_0023404	CCTCATCCTCATCGCAACCT	ACCCTCCATTGCTCTTCTGGA
miR‐136	ACACTCCAGCTGGGACTCCATTTGTTTT	CCAGTGCAGGGTCCGAGGT
IL‐6R	TTGTTTGTGAGTGGGGTCCT	TGGGACTCCTGGGAATACTG
IL‐1β	CAGAAGTACCTGAGCTCGCC	AGATTCGTAGCTGGATGCCG
IL‐6	GACTGATGTTGTTGACAGCCACTGC	AGCCACTCCTTCTGTGACTCTAACT
TNF‐α	CATGATCCGAGATGTGGAACTGGC	CTGGCTCAGCCACTCCAGC

### 2.7 Western blot analysis

To obtain the total proteins, RIPA lysis buffer added with PMSF was used at 4°C. The supernatant was obtained after cells were centrifuged at 9500 *g*/min for 5 minutes. Afterwards, BCA Protein Assay Kit (Beyotime, Shanghai, China) was carried out to measure protein concentration and the protein was boiled for degeneration. Then, the proteins were loaded on 10% SDS‐PAGE and transferred to PVDF membranes. We added the primary antibodies to the membranes for a whole night at 4°C. Primary antibodies, anti‐IL‐6R and anti‐GAPDH (1:1000, Abcam, Cambridge, UK), were used. After incubated with secondary antibodies for 2 hours, the membranes were exposed to the enhanced chemiluminescence reagent. Finally, the bands were evaluated utilizing ImageJ software.

### 2.8 Luciferase reporter assay

https://circinteractome.nia.nih.gov/ predicted circ_0023404 may be linked with miR‐136. The starBase software (http://starbase.sysu.edu.cn) predicted that IL‐6R may be combined with miR‐186. WT sequence of circ_0023404 and WT 3ʹUTR sequence of IL‐6R with the binding sites of miR‐136 was subcloned into the pGL3 Basic reporter vectors (Promega, Madison, WI, USA). To construct MUT sequence, the site‐directed mutagenesis was carried out using Quick Change Lightning kit (Stratagene, La Jolla, CA, USA). Briefly, cells were transfected with pGL3‐circ_0023404 WT/MUT or pGL3‐IL‐6R WT/MUT 3ʹUTR, together with miR‐136 mimics. Dual‐Luciferase Assay System (Promega, Madison, WI, USA) was used to analyse the luciferase activity.

### 2.9 ELISA

IL‐1β, IL‐6 and TNF‐α concentrations in the supernatants of HK‐2 cell cultures were determined using the corresponding ELISA Kit (Solarbio, Beijing, China). A microplate reader (Thermo Fisher Scientific; MA, USA) was utilized to record the OD values of samples at 450‐nm wavelength. Then, the regression equation was used to OD values to calculate the concentration of the samples.

### 2.10 ROS, MDA and SOD assay

Following transfection and H/R induction for 48 hours, HK‐2 cells in the DMEM medium were centrifuged at 4000 *g* for 10 minutes to collect the cell supernatant used to measure the contents of ROS, MDA and SOD. An ROS Assay Kit (Beyotime, Shanghai, China) was used to test ROS accumulation. A Lipid Peroxidation MDA Assay Kit (Beyotime, Shanghai, China) and a SOD Activity Assay Kit (BioVision, Milpitas, CA, USA) were conducted to tested MDA and SOD content.

### 2.11 Statistical analysis

Three independent experiments were performed, and experimental data were expressed as mean ± standard deviation. Statistical analysis was executed using GraphPad 6.0 software. We calculated *P*‐value using Student's *t* test or ANOVA. A *P* < .05 was indicated to be statistically significant.

## 3 RESULTS

### 3.1 Expression of circ_0023404 in serum of AKI patients and H/R‐incubated HK‐2 cells

**FIGURE 1:**
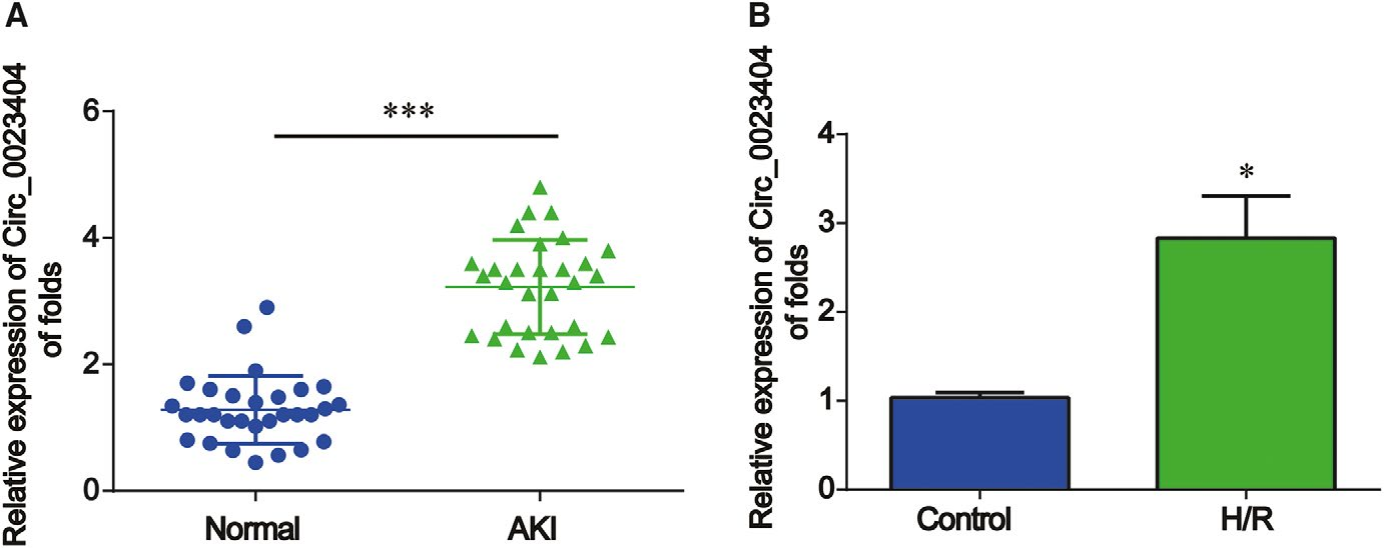
The relative expression levels of circ_0023404 in AKI patients and H/R‐treated HK‐2 cells were increased. A, circ_0023404 expression levels were elevated in AKI serum samples than that in normal serum samples. RT‐qPCR was carried out to evaluate circ_0023404 expression in serum samples of patients with AKI (n = 30) and healthy cases (n = 30). B, HK‐2 cells were exposed to H/R condition. Circ_0023404 expression was detected using RT‐qPCR. Three independent experiments were carried out. Error bars stand for the mean ± SD of at least triplicate experiments. *P < .05, ***P < .001

Firstly, the relative expression level of circ_0023404 was determined by real‐time qPCR in a total of 30 AKI patients. In Figure 1A, circ_0023404 expression was greatly increased in AKI patients. Then, we investigated the effect of H/R condition on HK‐2 cells. HK‐2 cells were exposed to H/R environment to set up AKI cell model. In Figure 1B, we displayed that circ_0023404 was up‐regulated in H/R‐treated cells. These implied that the expression of circ_0023404 was increased in AKI patients and in HK‐2 cells treated with H/R condition.

### 3.2 Loss of circ_0023404 reduced H/R‐triggered injury in HK‐2 cells

**FIGURE 2:**
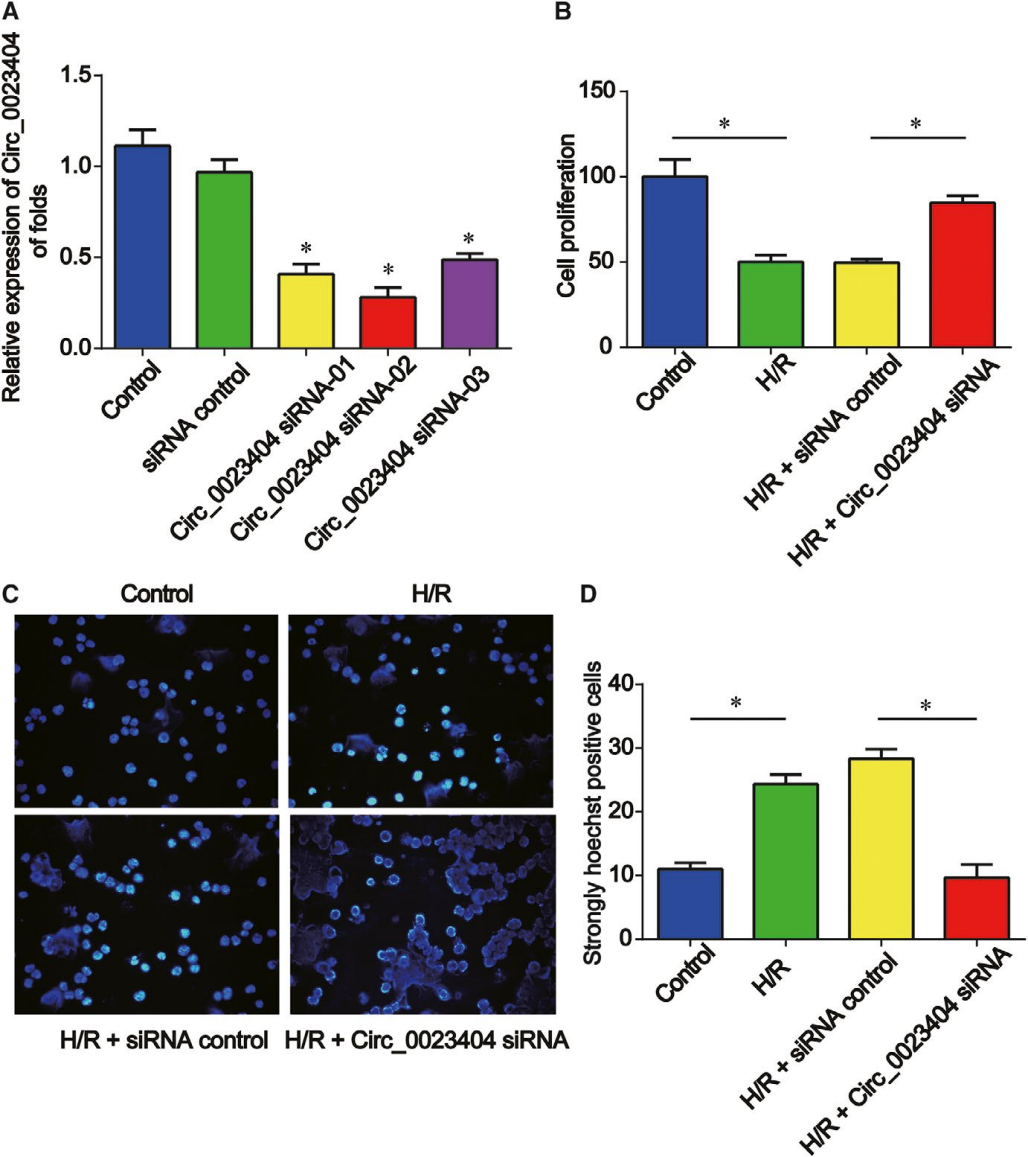
Loss of circ_0023404 increased HK‐2 cells proliferation and inhibited cell apoptosis. A, After transfection with circ_0023404 siRNA, the transfection efficiency was evaluated using RT‐qPCR. HK‐2 cells were transfected with circ_0023404 siRNA and then exposed to H/R environment. H/R (B) Cell viability was tested by CCK‐8 assay. C and D, Cell apoptosis ratio was assessed by Hoechst 33342 assay. Three independent experiments were carried out. Error bars stand for the mean ± SD of at least triplicate experiments. *P < .05

Next, we investigated whether circ_0023404 participated in HK‐2 cell processes. Circ_0023404 siRNA was transfected to modulate circ_0023404 expression in HK‐2 cells. It was confirmed circ_0023404 was significantly decreased by circ_0023404 siRNA and circ_0023404 siRNA‐02 exerted a best effect in HK‐2 cells (Figure 2A). Then, circ_0023404 siRNA‐02 was used in subsequent assays. After exposed to H/R treatment, cell viability and apoptosis were determined. In Figure 2B, we found H/R exposure significantly reduced HK‐2 cell viability. HK‐2 cell growth was induced by circ_0023404 siRNA. In addition, H/R was able to induce cell apoptosis, which was prohibited by loss of circ_0023404 in vitro (Figure 2C and D).

### 3.3 Down‐regulation of circ_0023404 repressed inflammatory response

**FIGURE 3:**
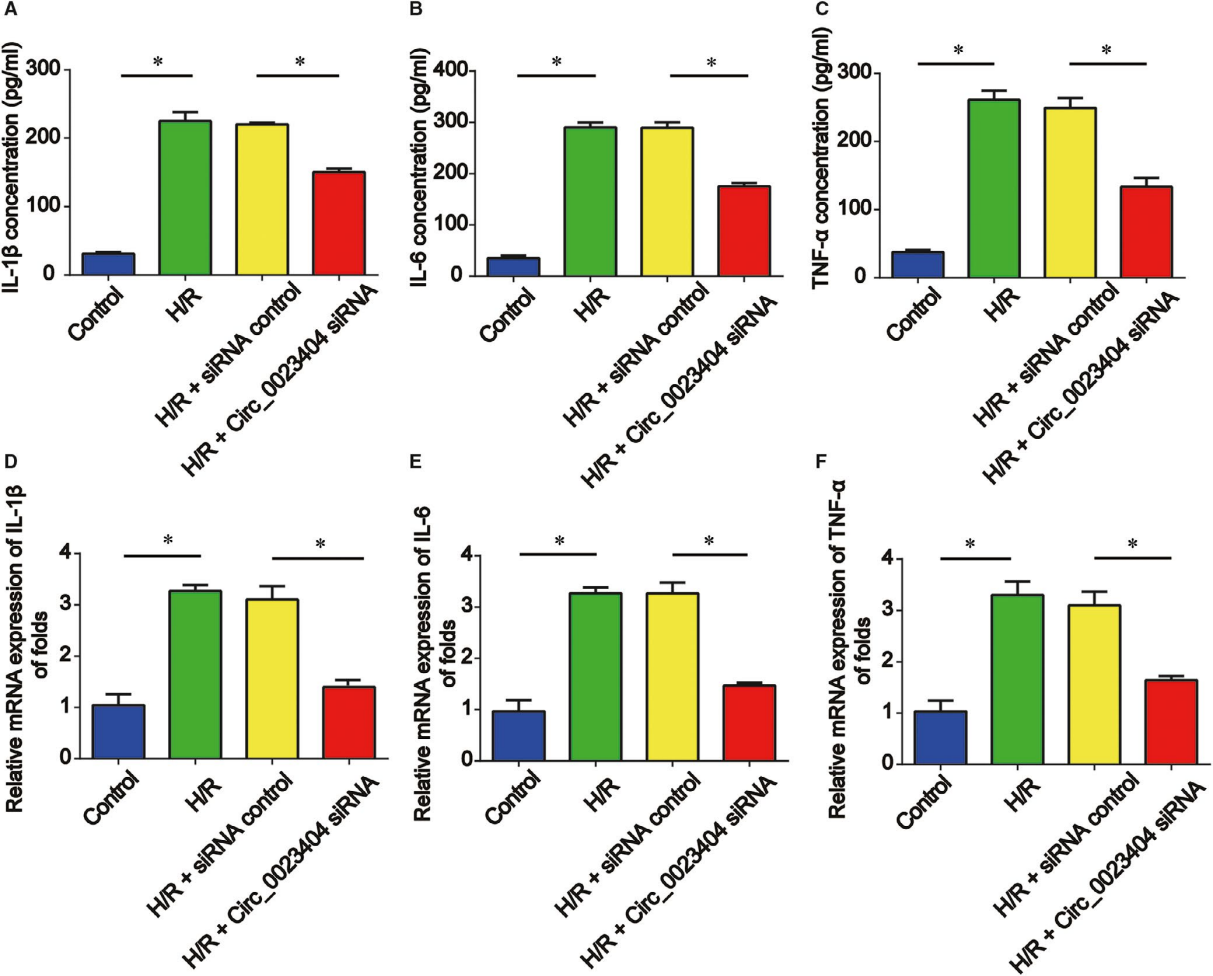
Decrease of circ_0023404 repressed the secretion of inflammatory cytokines in HK‐2 cells under H/R environment. HK‐2 cells transfected with circ_0023404 siRNA and then subjected to H/R treatment. A, B and C, ELISA was employed to examine the concentrations of inflammatory cytokines (IL‐1β, IL‐6 and TNF‐α). D, E and F, RT‐qPCR assay was carried out to examine the mRNA expression of IL‐1β, IL‐6 and TNF‐α. Three independent experiments were carried out. Error bars stand for the mean ± SD of at least triplicate experiments. *P < .05

Meanwhile, cell inflammatory response was assessed by detecting inflammatory cytokines. Whether circ_0023404 was involved in inflammation of HK‐2 cells under H/R was investigated. We observed that IL‐1β, IL‐6 and TNF‐α protein expression in HK‐2 cell culture medium could be triggered by H/R treatment. Decrease of circ_0023404 was able to reverse this process (Figure 3A‐C). Additionally, IL‐1β, IL‐6 and TNF‐α mRNA levels extracted from HK‐2 cells demonstrated a similar tendency as shown in Figure 3D‐F.

### 3.4 Decrease of circ_0023404 reduced H/R‐triggered oxidative stress of HK‐2 cells

**FIGURE 4:**
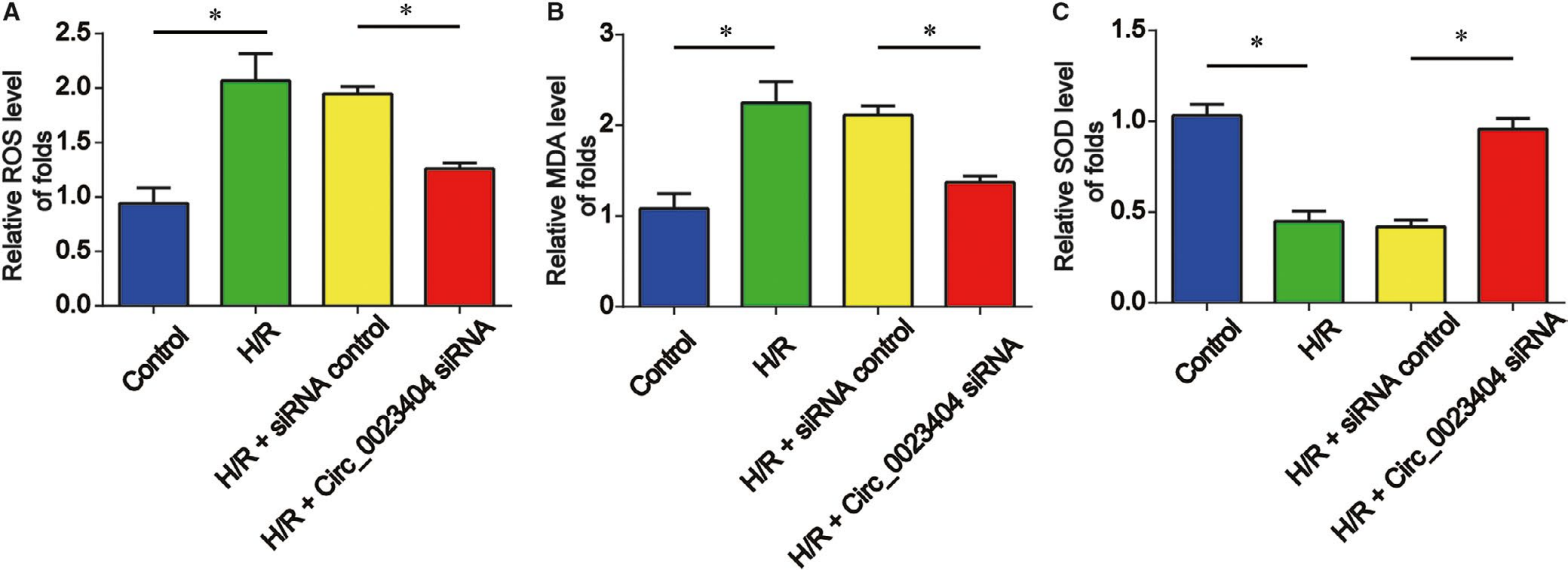
Down‐regulation of circ_0023404 repressed oxidative stress in HK‐2 cells under H/R stimulation. HK‐2 cells transfected with circ_0023404 siRNA and then subjected to H/R treatment. A, ROS generation was evaluated by ROS assay. B, MDA content was determined by a MDA Assay Kit. C, SOD activity was determined by a SOD Activity Assay Kit. Three independent experiments were carried out. Error bars stand for the mean ± SD of at least triplicate experiments. *P < .05

Next, we further determined whether circ_0023404 regulated oxidative stress in HK‐2 cells. We displayed H/R treatment strongly upgraded ROS generation, MDA contents and increased SOD activity (Figure 4A‐C). For another, loss of circ_0023404 was able to repress the oxidative stress process as manifested in Figure 4A‐C.

### 3.5 Circ_0023404 sponged miR‐136

**FIGURE 5:**
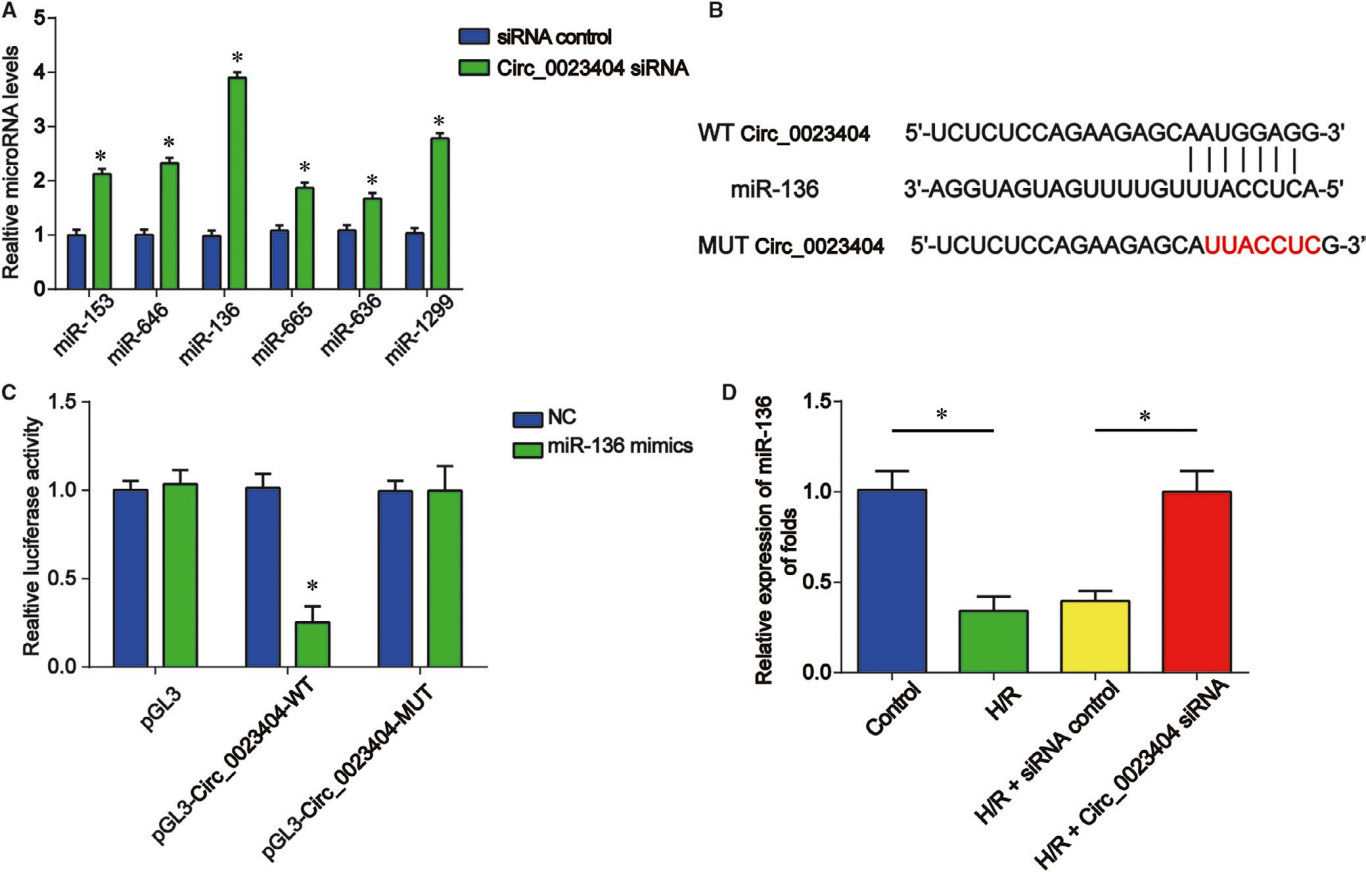
Circ_0023404 acted as a sponge for miR‐136. A, HK‐2 cells were transfected with circ_0023404 siRNA or siRNA control and then stimulated in H/R environment. miRNA expression was tested by RT‐qPCR. B, Schematic diagram of predicted binding sites of circ_0023404 with miR‐136. C, The luciferase reporter constructs containing the wild‐type (WT‐circ_0023404) or mutant circ_0023404 (MUT‐circ_0023404). The correlation between them was assessed by dual‐luciferase reporter assay. WT‐circ_0023404 or MUT‐circ_0023404 was co‐transfected with miR‐136 mimics or their corresponding negative controls. D, HK‐2 cells were transfected with circ_0023404 siRNA or siRNA control and then stimulated in H/R environment. The miR‐136 expression was detected by RT‐qPCR. Three independent experiments were carried out. Error bars stand for the mean ± SD of at least triplicate experiments. *P < .05

**FIGURE 6:**
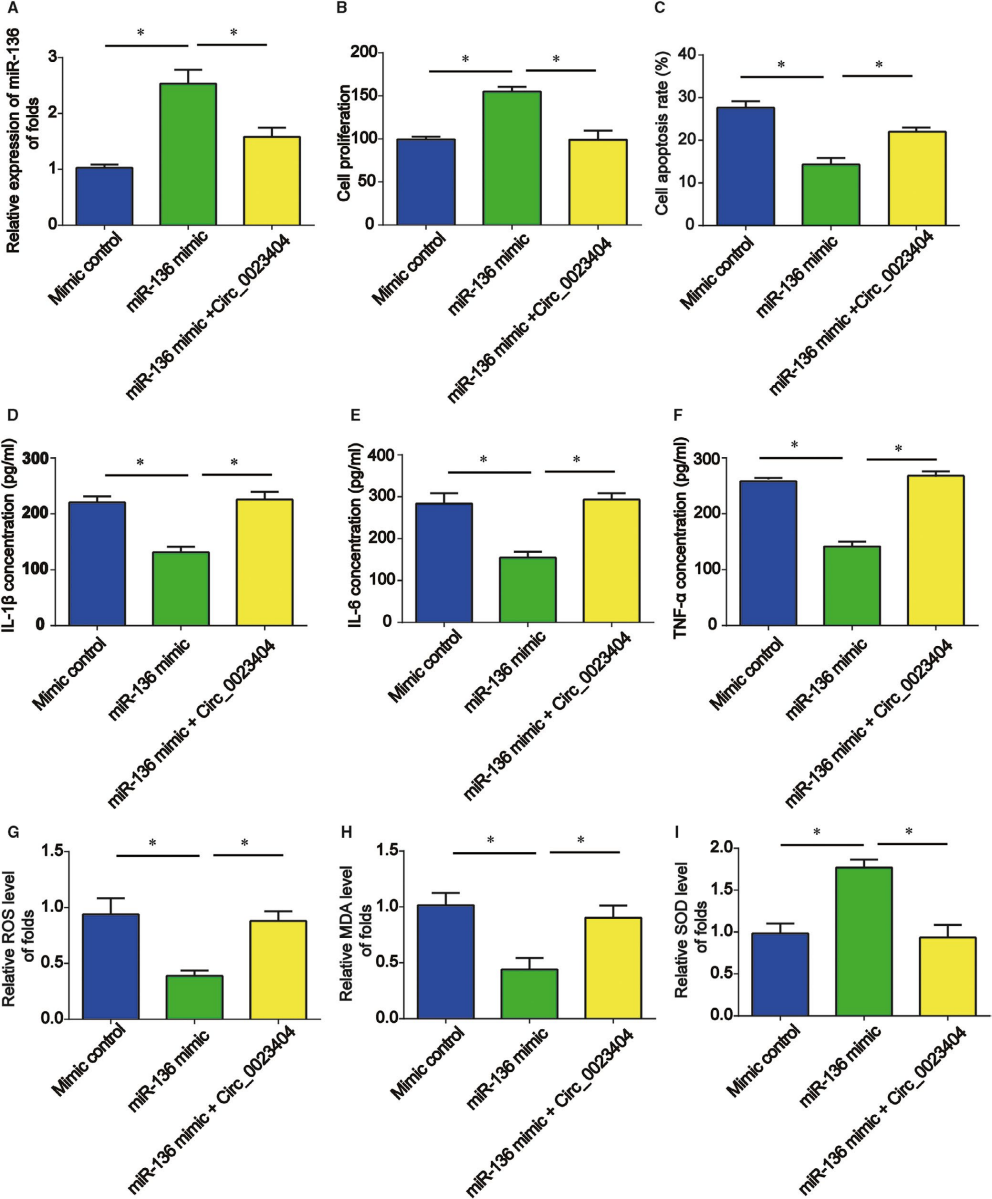
Circ_0023404 contributed to H/R‐caused injury via down‐regulating miR‐136. A, miR‐136 expression. After transfection with miR‐136 mimic or NC mimic, HK‐2 cells were transfected with pcDNA‐circ_0023404 and then indicated in H/R condition. B, Cell viability was tested by CCK‐8 assay. C, Cell apoptosis ratio was assessed by Hoechst 33342 assay. D, E and F, ELISA was used to examine the concentrations of inflammatory cytokines (IL‐1β, IL‐6 and TNF‐α). G, H and I, ROS generation, MDA content and SOD activity. Three independent experiments were carried out. Error bars stand for the mean ± SD of at least triplicate experiments. *P < .05

Moreover, bioinformatical analysis (https://circinteractome.nia.nih.gov/) was employed to predict the microRNA targets of circ_0023404. HK‐2 cells were transfected with circ_0023404 siRNA and then subjected to H/R treatment. Next, RT‐qPCR was carried out to assess miRNA expression. In Figures 5A, 6 miRNAs (miR‐153, miR‐646, miR‐136, miR‐665, miR‐636 and miR‐1299) were apparently induced by silencing circ_0023404. miR‐136 expression was changed most significantly. miR‐136 was selected miR‐136 in our following research. The binding sites between miR‐136 and circ_0023404 are shown in Figure 5B. Then, MUT and WT sequence of circ_0023404 were cloned into pGL3 vector to construct circ_0023404 MUT and circ_0023404 WT plasmids. Luciferase reporter assay revealed that the luciferase activity was decreased in cells co‐transfected with circ_0023404 WT and miR‐136 mimics in Figure 5C. As displayed in Figure 5D, miR‐136 was negatively regulated by circ_0023404 in HK‐2 cells.

### 3.6 Circ_0023404 contributed to HK‐2 cell injury caused by H/R via sponging miR‐136

Furthermore, miR‐136 mimics were used to enhance miR‐136 expression in HK‐2 cells. In Figure 6A, miR‐136 was greatly induced by miR‐136 mimics, which was reversed by circ_0023404 overexpression plasmid. Transfection with circ_0023404 overexpressing plasmid and miR‐136 mimics reduced HK‐2 cell viability as displayed in Figure 6B. In addition, in Figure 6C, an enhancement of cell apoptosis was triggered by circ_0023404 up‐regulation in vitro. Consistently, we revealed that miR‐136 reduced inflammatory cytokines and oxidative stress, which could be enhanced by overexpression of circ_0023404 as demonstrated in Figure 6D‐I.

### 3.7 IL‐6R was a target of miR‐136

**FIGURE 7:**
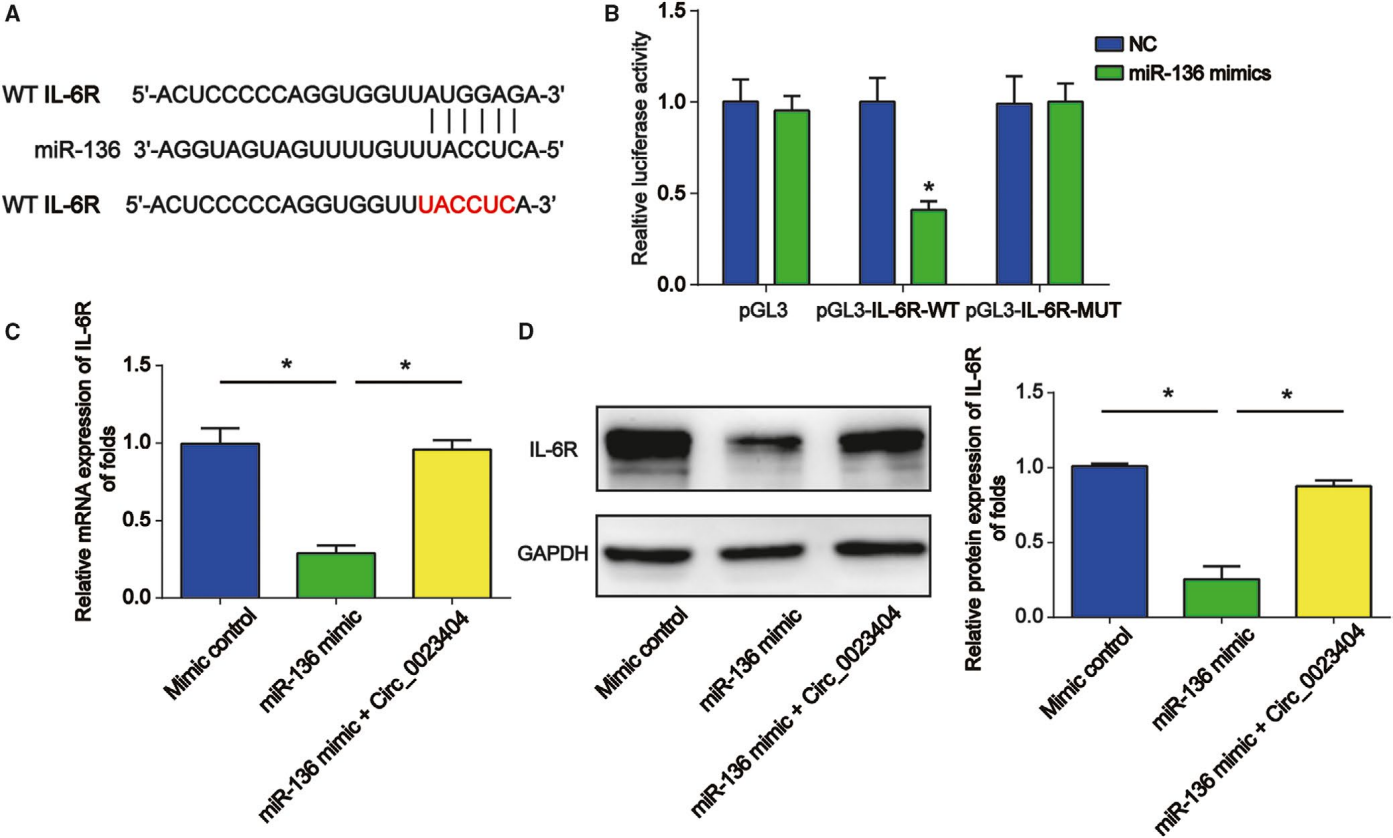
IL‐6R acted as a downstream target for miR‐136. A, Schematic diagram of predicted binding sites of IL‐6R with miR‐136. B, The luciferase reporter constructs containing the wild‐type (WT‐IL‐6R) or mutant IL‐6R (MUT‐IL‐6R). The correlation between them was assessed by dual‐luciferase reporter assay. C and D, IL‐6R mRNA and protein expression in HK‐2 cells. HK‐2 cells were transfected with miR‐136 mimic and pcDNA‐circ_0023404 for 48 h and then exposed to H/R condition. Three independent experiments were carried out. Error bars stand for the mean ± SD of at least triplicate experiments. *P < .05

Then, http://starbase.sysu.edu.cn/ was used to predict the link between miR‐136 and IL‐6R. In Figure 7A, the binding regions between miR‐136 and IL‐6R were exhibited. In addition, we constructed luciferase reporter plasmids of WT‐IL‐6R and MUT‐IL‐6R. Co‐transfection of WT‐IL‐6R with miR‐136 mimics significantly reduced the reporter activity (Figure 7B). Besides these, miR‐136 mimics greatly repressed IL‐6R mRNA and protein, which was rescued by overexpression of circ_0023404 in Figure 7C and D.

## 4 DISCUSSION

Acute kidney injury is characterized by sharply renal dysfunction, and it is a common complication in hospitalized patients.^16^ Here, in our work, we observed that circ_0023404 was up‐regulated in AKI patients and H/R‐incubated HK‐2 cells. Loss of circ_0023404 reduced cell injury via expediting cell growth, repressing cell apoptosis, decreasing inflammatory factor secretion and oxidative stress generation HK‐2 cells stimulated by H/R. In addition, circ_0023404 served as a sponge of miR‐136. Next, miR‐136 overexpression exhibited a repressive impact on cell injury. Moreover, IL‐6R was a target of miR‐136. Overexpression of circ_0023404 induced IL‐6R expression via sponging miR‐136 in HK‐2 cells under H/R. To our knowledge, this is the first report of circ_0023404 being involved in the development of AKI.

In recent years, circRNAs have been known as crucial regulators in many disease, including AKI.^17^, ^18^ For example, circRNA‐YAP1 can act as the sponge of miR‐21‐5p to protect HK‐2 cell injury activated by I/R.^19^ Circ_0114427 can participate in the modulation of AKI.^20^ Circ_0023404 is identified as a novel master in several cancers. Circ_0023404 can promote lung cancer development via regulating miR‐217 and ZEB1.^21^ Circ_0023404 can promote cervical cancer via modulating miR‐136, TFCP2 and YAP signalling.^22^ In addition, circ_0023404 induces cervical cancer development via modulating VEGFA through sponging miR‐5047.^23^ However, the role of circ_0023404 underlying AKI is still unclear. Currently, we found that circ_0023404 was obviously increased in AKI patients and HK‐2 cells under H/R environment. I/R‐triggered injury is a major reason which induces the onset of AKI.^24^ I/R injury can contribute to inflammatory cytokines releases and cell apoptosis in HK‐2 cells.^25^ Here, we reported that loss of circ_0023404 reduced HK‐2 cell apoptosis, induced cell proliferation and repressed the secretion of inflammation cytokines and oxidative stress under H/R condition.

During the past few decades, the association between circRNAs and microRNAs has been widely reported.^26^, ^27^ CircRNAs act as significant sponges of microRNAs, and they can reduce microRNA activity. Furthermore, the importance of microRNAs in AKI process has been focused on these years. Here, miR‐136 was predicted as a target for circ_0023404. Many studies show that miR‐136 participates in various types of cancers.^28^, ^29^, ^30^ In addition, miR‐136 has been shown to improve renal fibrosis in diabetic rats through down‐regulation of tyrosine kinase SYK and TGF‐β1/Smad3 pathway.^31^ Here, it was displayed that circ_0023404 could sponge miR‐136. Overexpression of miR‐136 reduced HK‐2 cell injury triggered by H/R condition. All these data hinted that circ_0023404 might result in HK‐2 cell injury through sponging miR‐136. However, the molecular function of circ_0023404 and its other potential downstream miRNA target genes in AKI pathogenesis need to be fully investigated.

IL‐6 exhibits significant pro‐ or anti‐inflammatory capacity.^32^, ^33^ IL‐6 functions via binding with IL‐6R, which can activate Jak/STAT signalling pathway.^34^ Additionally, IL‐6/IL‐6R has been reported to exert a crucial role in AKI.^35^ In our present study, we predicted that IL‐6R was a target of miR‐136. In our data, we observed that circ_0023404 can regulate IL‐6R activation in HK‐2 cells under H/R through modulating miR‐136. Subsequently, we also demonstrated the direct correlation between them.

In general, this work indicated that circ_0023404 resulted in HK‐2 cells injury induced by H/R via sponging miR‐136 expression and inducing IL‐6R level. Advances in the understanding of circ_0023404‐mediated HK‐2 cell injury can provide some basic knowledge on the possible therapy for AKI.

## CONFLICT OF INTEREST

The authors confirm that there are no conflicts of interest.

## AUTHOR CONTRIBUTIONS

**Yong Xu:** Data curation (equal); Software (equal); Writing‐original draft (equal). **Xiang Li:** Formal analysis (equal); Supervision (equal). **Hailun Li:** Formal analysis (equal); Supervision (equal); Validation (equal). **Lili Zhong:** Investigation (equal); Visualization (equal). **Yongtao Lin:** Methodology (equal); Validation (equal); Visualization (equal). **Juan Xie:** Formal analysis (equal); Project administration (equal); Visualization (equal). **Donghui Zheng:** Conceptualization (lead); Resources (lead).

## Data Availability

The data that support the findings of this study are available from the corresponding author upon reasonable request.
